# Serum Protein Signatures for Breast Cancer Detection in Treatment-Naïve African American Women Using Integrated Proteomics and Pattern Analysis

**DOI:** 10.3390/s26020403

**Published:** 2026-01-08

**Authors:** Padma P. Tadi Uppala, Elmer C. Rivera, Hyun J. Kwon, Sharon S. Lum

**Affiliations:** 1 School of Population Health, Nutrition & Wellness, Andrews University, Berrien Springs, MI 49104, USA; 2 Department of Engineering, Andrews University, Berrien Springs, MI 49104, USA; elmeralbertocr@gmail.com (E.C.R.); hkwon@andrews.edu (H.J.K.); 3 Department of Surgery, Loma Linda University Health, Loma Linda, CA 92354, USA; slum@llu.edu

**Keywords:** breast cancer, serum proteomics, protein biomarkers, 2D-DIGE, MALDI-TOF/TOF, LC–MS/MS, proteomic sensor platform, cumulative distribution function, random forest, treatment-naïve African American women

## Abstract

Breast cancer is the leading cause of cancer-related mortality in African American (AA) women. In this study we evaluated the serum proteomic profile of AA women with breast cancer using an integrated proteomic framework with multivariate pattern analysis. Using 2D-DIGE, thousands of serum protein spots were detected across 33 gels; 46 spots met criteria for presence, statistical significance, and differential expression. Proteins from the spots were identified by MALDI-TOF/TOF and matched in curated databases, highlighting serum biomarkers including ceruloplasmin, alpha-2-macroglobulin, complement component C3 and C6, alpha-1-antitrypsin, alpha-1B-glycoprotein, alpha-2-HS-glycoprotein and haptoglobin-related protein. LC–MS/MS analysis revealed 163 differentiating peptides after imputing and filtering 286 peptides. These were evaluated using cumulative distribution function (CDF) analysis, a nonparametric method suited for limited sample sizes. Peptide patterns were explored with Random Forest, showing concordance with CDF. The model achieved an AUC of 0.85 at the peptide level. This workflow identified differentiating proteins (CERU, A2MG, CO3, VTDB, HEMO, APOB, APOA4, CFAH, CO4A, AACT, K1C10, ITIH2, ITIH4), highlighting CERU, A2MG, and CO3 with overexpression and reproducible identification across platforms. We present an integrated, non-invasive serum protein biomarker signature panel specific to AA women, through reproducible proteomic sensor framework to support early detection and breast cancer prevention.

## 1. Introduction

Breast cancer is the most common cancer among women worldwide (World Health Organization, 2024) and remains the second leading cause of cancer-related death among women in the United States [1]. For 2025, an estimated 316,950 new invasive cases, 59,250 cases of ductal carcinoma in situ (DCIS), and 42,170 deaths are expected among U.S. women [2]. Although incidence rates are lower among non-Hispanic (NH) Black women when compared with White women (128 vs. 133 per 100,000), mortality is significantly higher among NH Black women (26.4 vs. 19.4 per 100,000) [1,3]. These racial disparities likely stem from the complex interplay of tumor biology, genetic predisposition, access to medical care, and socioeconomic factors [4]. African American women are more often diagnosed at a younger age and with aggressive breast cancer subtypes, particularly basal-like and triple-negative breast cancers (TNBC), and a subset carries BRCA1 or BRCA2 mutations [5,6,7,8]. TNBC is associated with high metastatic potential, poor response to hormonal therapy, recurrence, and worse prognosis [9].

Current evidence on serum protein biomarkers of breast cancer across different ethnic groups, including African American women, remains limited, highlighting the need to evaluate variability in circulating proteomic profiles across populations [10,11]. Tumor-based studies have identified significant differences between African American and Caucasian women, including variations in gene expression and molecular subtype distribution. For example, African American women show a higher prevalence of basal-like or triple-negative breast cancers, and differences in signaling pathways may influence protein release into circulation, providing biological mechanisms linking tumor heterogeneity to serum proteomic profiles [12,13]. Sociodemographic factors and differential access to healthcare further contribute to disparities in breast cancer diagnosis and outcomes, underscoring the importance of accounting for ethnic variability in serum biomarker evaluation [12].

In breast cancer patients, serum proteomic profiles show marked alterations compared with healthy controls, including increased levels of inter-alpha trypsin inhibitor heavy chain 4 (ITIH4) fragments and C3a-desArg prior to clinical diagnosis, consistent with early inflammatory and acute-phase responses. Alterations in additional serum proteins, such as alpha-2-macroglobulin, ceruloplasmin, afamin, and apolipoprotein E, further reflect dysregulation of immune response, lipid metabolism, and protease activity associated with tumor presence [14]. Importantly, blood-based studies have demonstrated that breast cancer-associated protein alterations are detectable in serum and that race-specific differences in circulating protein expression have been reported between African American and Caucasian women, reinforcing the relevance of serum biomarkers for investigating breast cancer disparities [15,16]. These observations collectively support the need to validate serum protein panels in ethnically diverse cohorts.

Despite advances in imaging and biopsy techniques, including BI-RADS classification and histopathological confirmation, challenges such as false positives, false negatives, and overdiagnosis continue to persist [17,18]. Therefore, complementary and minimally invasive diagnostic tools are needed. Proteomics-based strategies have emerged as promising solutions: approaches such as 2D-DIGE combined with MALDI-TOF/TOF enable high-resolution and reliable detection of differentially expressed serum proteins [19], while LC–MS/MS provides a deep and quantitative peptide-level profile [20].

Mass spectrometry-based proteomics is a highly sensitive tool that has identified numerous clinically relevant biomarkers across diverse contexts, including urinary proteomics [21], head and neck cancer [22], pancreas [23], bladder [24] and breast [25]. While serum proteomics studies have identified numerous candidate biomarkers for breast cancer, these range from early-stage panels to proteins associated with metastatic progression [26,27]. Key limitations include the low specificity of many candidates, which often reflect systemic inflammatory processes rather than tumor-specific alterations, as well as the challenge of translating these candidates into clinically useful biomarkers [28]. Moreover, reproducibility across cohorts and technological platforms has been inconsistent, and many proposed panels lack rigorous independent and prospective validation [29]. A critical gap in this field is the systematic underrepresentation of diverse populations, particularly African American women, in biomarker discovery and validation studies [30]. Given that this population exhibits a higher incidence of aggressive breast cancer subtypes and increased mortality, it is crucial to evaluate the generalizability of existing serum proteomic profiles and to identify potential signatures specific to clinical disparities. Our study addresses this need through quantitative proteomic profiling in a well-characterized cohort with a substantial proportion of African American women, aiming to identify and validate serum protein signatures that overcome the limitations of current biomarkers. Proteomic profiling in African American women has revealed distinct serum patterns differentiating cancer patients from healthy controls, supporting the use of multi-protein serum panels rather than single biomarkers for early detection [5,6]. Therefore, developing methods capable of discriminating cancer-specific proteomic signatures in blood serum represents a promising framework for early diagnosis and disease monitoring. Reliance on individual proteins, rather than multicomponent signatures may lead to reduced diagnostic specificity.

Advanced computational and statistical approaches have become essential in proteomic data analysis, enabling the identification of subtle patterns, robust handling of complex datasets, and accurate biomarker discovery. Among these, machine learning (ML) algorithms, such as Random Forest, are particularly valuable for modeling complex and non-linear relationships, identifying patterns within large datasets, and generating predictive models without assuming parametric distributions [31,32,33]. However, most existing studies rely on single-population cohorts, highlighting the need to include vulnerable and diverse populations to ensure that findings are broadly applicable [6]. Cumulative distribution function (CDF) analysis has demonstrated effectiveness across diverse contexts, including sparse, skewed, or outlier-rich datasets [34], and is particularly well-suited for the complex data structures generated in proteomics [35]. Finally, imputation techniques are essential for addressing missing values, which are common in LC–MS/MS datasets due to low-abundance peptides, stochastic sampling, or technical variability. Modern approaches, such as autoencoders and collaborative filtering, have proven effective in recovering missing information while preserving variance and protein–protein correlations, ensuring that downstream analyses, whether ML- or CDF-based remain robust, unbiased, and capable of detecting biologically meaningful biomarker patterns [36].

Building on these advances, our study implements an integrated, multivariate, multi-method framework for the identification of serum biomarkers of early-stage breast cancer in treatment-naïve African American women. Complementary proteomic techniques were employed, including 2D-DIGE for the detection of differentially expressed proteins, MALDI-TOF/TOF for protein identification, and LC–MS/MS for high-resolution peptide profiling, collectively functioning as a multi-channel measurement platform for serum analysis. Data were processed using rigorous imputation and filtering methods to account for missing or low-abundance values, followed by non-parametric cumulative distribution function (CDF) analysis and Random Forest modeling to prioritize the most informative proteomic patterns. This multivariate, multi-method approach constitutes a reproducible and potentially generalizable proteomic sensing platform, with promising applications in early detection, clinical monitoring, and precision medicine for vulnerable and high-risk populations.

## 2. Materials and Methods

**Figure 1:**
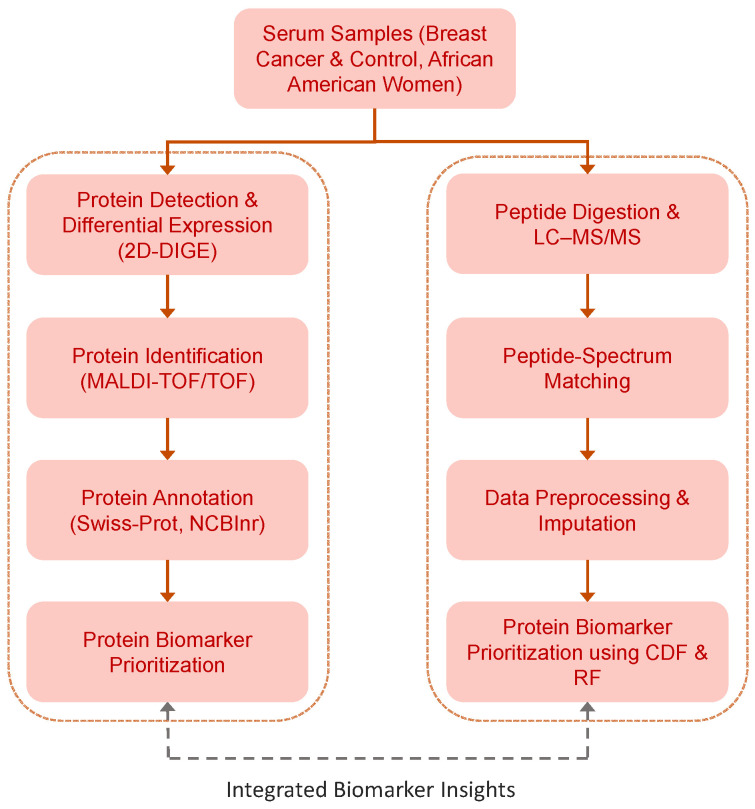
Integrated serum proteomics workflow for biomarker identification and evaluation in African American women with breast cancer. Serum samples were analyzed by MALDI-TOF/TOF and LC–MS/MS for protein biomarker prioritization; LC–MS/MS workflow performed protein prioritization from peptide-level data using advanced algorithms such as the cumulative distribution function (CDF) and Random Forest (RF). Both approaches converge, consolidating results and enabling robust detection, quantification, and validation of disease-associated proteins and peptides.

This study implemented a serum proteomics approach to identify potential protein biomarkers for breast cancer in African American women (Figure 1). Serum samples were processed to remove high-abundance proteins and subjected to complementary analyses. Gel-based proteomics (2D-DIGE) enabled the detection and comparison of differentially expressed proteins, followed by Protein Identification via MALDI-TOF/TOF and Protein Annotation (Swiss-Prot, Swiss Institute of Bioinformatics [SIB], Lausanne, Switzerland; NCBInr, National Center for Biotechnology Information [NCBI], Bethesda, MD, USA), which performed protein biomarker prioritization. Multidimensional LC–MS/MS proteomics provided peptide-level data that were preprocessed, imputed, and aggregated for multivariate pattern analysis. Protein biomarker prioritization was conducted from LC–MS/MS peptide-level data using advanced algorithms such as the cumulative distribution function (CDF) and Random Forest (RF), considering the cumulative evidence of differentiating peptides associated with each protein. This integrated framework enabled the detection, quantification, and validation of peptides and proteins associated with breast cancer, consolidating findings from both approaches into a unified panel of protein biomarkers with potential for diagnostics and providing a solid foundation for subsequent clinical and functional studies.

### 2.1. Patient Cohorts and Serum Sample Preparation

Human serum samples were obtained from treatment-naïve breast cancer patients and healthy control individuals for two complementary proteomic approaches. For the Gel-based Proteomic Analysis (2D-DIGE/MALDI-TOF/TOF), serum samples from 11 breast cancer patients (Group 1) and 11 healthy controls (Group 2) were used. The breast cancer cohort was consisting of Black or African American ethnicity and included both pre- and post-menopausal women. Tumor sub-types encompassed invasive/atypical carcinoma (IN/AC), invasive/ductal carcinoma (IN/IDC), infiltrating ductal carcinoma (IDC), and ductal carcinoma in situ (DCIS), with diverse hormone receptor profiles including ER+/PR+, ER−/PR−, HER2+, and ER−/PR−/HER2−. The control cohort consisted of healthy women with no history of cancer. For the Serum Proteomics by MudPIT (Multidimensional Protein Identification Technology, LC/MS/MS), a subset of 6 breast cancer patients and 6 matched healthy controls with similar demographic and clinical characteristics was analyzed. For both approaches, human serum samples were processed using the Multiple Affinity Removal System (MARS; Agilent Technologies, Santa Clara, CA, USA) to deplete high-abundance proteins, enhancing the detection of lower-abundance proteins in downstream proteomic analyses.

The clinicopathological characteristics of the gel-based 2D-DIGE and LC/MS/MS cohorts were recorded in detail. Breast cancer patients ranged in age from 29 to 74 years, with BMI values between 20 and 43 kg/m^2^, and included both pre- and post-menopausal women. Tumor subtypes comprised invasive neoplasia (IN), invasive neoplasia/adenocarcinoma (IN/AC), mixed invasive and ductal carcinoma in situ (IN/IDC), invasive ductal carcinoma (IDC), and ductal carcinoma in situ (DCIS). Disease stages spanned 0–4, including sub-stages IIIA, IIB and IIIC, with tumor grades ranging from low to high and lymph node status varying between N− and N+. Hormone receptor profiles included ER+/PR+, ER−/PR−, HER2+, and HER2−, and receptor types were classified as Luminal A, HER2-positive, or triple-negative. Healthy controls ranged in age from 20 to 67 years, with BMI values between 19 and 32 kg/m^2^, and included both pre- and post-menopausal women.

### 2.2. Gel-Based Proteomic Analysis (2D-DIGE)

#### 2.2.1. Fluorescent Labeling and 2D Electrophoresis

Protein concentrations of MARS-depleted serum samples were determined using the Bradford assay. For each analytical gel, 50 µg of protein from two individual samples and an internal standard were minimally labeled with CyDye DIGE fluors and multiplexed prior to electrophoresis. Breast cancer and control samples were randomly labeled with Cy3 or Cy5, while the internal standard, prepared by pooling equal amounts of all samples, was labeled with Cy2 and included on every gel for cross-gel normalization. Labeled samples were mixed and focused on first-dimension IPG strips (pH 4–7, non-linear) for a total of 65 kVh at 20 °C. Prior to SDS-PAGE, the IPG strips were equilibrated with equilibration buffer for 15 min, followed by another 10 min equilibration with the same buffer substituting iodoacetamide for DTT. The strips were then loaded onto 24 × 20 cm SDS-PAGE gels (exponential 4–20% gradient) and run in parallel using a DALT 12 electrophoresis system at 2 W per gel, 20 °C.

#### 2.2.2. Image Acquisition and Differential Analysis

The gels were scanned using a Typhoon 9400 imager (GE Healthcare, Chicago, IL, USA) to visualize the fluorescently labeled proteins. CyDye-labeled images were acquired with a 488 nm laser and a 520 nm band-pass (BP 40) emission filter for Cy2, a 532 nm laser and a 580 nm BP30 emission filter for Cy3, and a 633 nm laser and a 670 nm BP30 emission filter for Cy5. All gels were scanned at a resolution of 100 µm, and the photomultiplier tube (PMT) voltage for each channel was adjusted to prevent signal saturation during image acquisition. Image analysis was performed using the DeCyder v5.02 software (GE Healthcare, Chicago, IL, USA) for Differential In-Gel Analysis (DIA). Protein spots detected in paired gel images were co-analyzed in the DIA module, which included background subtraction, noise filtering, spot volume quantification, normalization, and volume ratio calculation. The DIA-processed spot maps, along with the corresponding original gel images, were subsequently imported into the Biological Variance Analysis (BVA) module for inter-gel spot matching and statistical evaluation of differentially expressed proteins. Protein spots with a Student *t*-test *p*-value < 0.05 were considered statistically significant. The magnitude of change was calculated and expressed as the Average Ratio (Group 2/Group 1).

#### 2.2.3. Spot Picking and In-Gel Digestion

Differentially expressed protein spots were selected for mass spectrometric identification. A preparative gel was run and stained with SyproRuby or Deep Purple dye. The image was acquired on a Typhoon 9400 (GE Healthcare, Chicago, IL, USA), and differentially expressed spots were mapped from the analytical DIGE images to the preparative gel image within the BVA module. An automated pick list was generated, and spots of interest were excised using an Ettan Spot Handler Workstation (SHW; GE Healthcare, Chicago, IL, USA). The excised gel plugs were subjected to in-gel digestion with trypsin, and peptides were extracted.

### 2.3. MALDI-TOF/TOF Analysis for Biomarker Identification

Peptide extracts from gel fragments were spotted onto a MALDI target and analyzed using an ABI 4800 MALDI-TOF/TOF analyzer (Applied Biosystems, Foster City, CA, USA). The resulting data were processed with the 4000 Series Explorer software (Applied Biosystems, Foster City, CA, USA), and the peptide mass fingerprints (PMFs) were searched against the NCBInr and Swiss-Prot databases for protein identification. NCBInr provides broad coverage, including predicted sequences and isoforms, whereas Swiss-Prot contains manually reviewed and experimentally validated entries. Proteins identified in both databases were considered reliable, and discrepancies were attributed to differences in sequence completeness, database curation, or nomenclature. Identification metrics included Hit Mass and Hit Score (Swiss-Prot) and ProteinMW, ProteinPI, ProteinScore, and PepCount (NCBInr), which allow validation of structural consistency and sequence coverage. Relative protein abundance between patient and control samples was assessed using the AV Ratio obtained from 2D DIGE, where positive and negative values indicate overexpression or underexpression, respectively.

### 2.4. Serum Proteomics by MudPIT (Multidimensional Protein Identification Technology), LC/MS/MS

MARS-depleted serum samples were processed using an IgY12 antibody column (Beckman) to remove the 12 most abundant serum proteins. The flow-through fraction was digested with trypsin prior to LC/MS analysis. Digested samples were analyzed in triplicate using a high-performance mass spectrometer (either LTQFT or LCQ Deca XP, Thermo Fisher Scientific, Waltham, MA, USA) coupled to an online Surveyor LC system equipped with an autosampler. Samples were loaded onto a trap column using the sample pump and subsequently eluted onto an analytical column for online two-dimensional liquid chromatography coupled to tandem mass spectrometry (2DLC/MS/MS). Peptides were separated into two dimensions: the first based on charge using strong cation exchange (SCX) chromatography, and the second based on hydrophobicity using reverse-phase (RP) chromatography. Fractionation was performed in several steps using a salt-step gradient followed by an RP gradient. For each step, peptides from the SCX column were eluted onto the RP column and analyzed by tandem MS.

MS data from all steps were processed using Bioworks software version 3.3.1 SP1 (Thermo Fisher Scientific, Waltham, MA, USA) to generate a multiconsensus report of protein identifications. Protein identifications were validated using Peptide Prophet, and peptide intensities measured in the LC/MS survey scans were used to derive quantitative information across the serum samples. For each detected peptide, molecular mass, retention time, and detection frequency across samples were recorded, allowing assessment of instrumental performance, signal coverage, and variability between subjects. Missing values were evaluated, and data preprocessing included filtering and imputation steps to ensure a consistent dataset for subsequent analyses using multivariate pattern analysis.

#### 2.4.1. LC–MS/MS Data Preprocessing and Imputation

Peptide intensity data obtained by LC–MS/MS were imported and preprocessed to ensure consistency and data quality. For subsequent analyses, only peptides with a sufficient number of valid measurements in both groups, control and cancer, were considered, ensuring that statistical analyses were based on representative data. Missing intensity values were imputed using the MinProb method [37], which assumes that missing values mainly correspond to low-abundance peptides. Imputation was performed for each sample independently, so that the distribution of intensities within each sample was preserved. For each column, the mean (μ) and standard deviation (σ) of the observed intensities were calculated, and missing values were replaced according to Equation (1):

(1) $$X_{\mathrm{imputed}}=\mu -\mathrm{downshift}\cdot \sigma +\mathrm{width}\;\times \sigma \times \epsilon ,\epsilon ∼N(0,1)$$

In this equation, downshift determines how far below the mean the imputed values are placed, width controls the spread of the generated values, and ε represents a random number drawn from a normal distribution, adding natural variability to the imputed values. This procedure preserves the overall distribution of observed intensities while realistically filling in missing values. To evaluate the effect of the imputation, histograms of log_2_-transformed peptide intensities were generated before and after imputation for each sample. These visualizations allowed inspection of how missing values were recovered without substantially altering the overall intensity profile. However, when a high proportion of values is missing, imputation may generate a secondary low-intensity population, producing bimodal distributions. This potential bifurcation in the distribution may indicate that the estimation of low-abundance intensities is compromised under conditions of extensive missing data, and therefore the observed patterns should be interpreted in this context [38].

Finally, peptides measured in multiple rows were collapsed into a single representative value per peptide by averaging the intensities, while retaining associated metadata (protein identifiers and peptide details) from the first occurrence. This workflow produced a clean, imputed, and summarized peptide intensity matrix suitable for subsequent quantitative and statistical analyses.

#### 2.4.2. Biomarker Identification from LC–MS/MS Data

For biomarker identification, peptide differentiation between cases and controls was evaluated using a complementary approach combining cumulative distribution function (CDF)-based analysis and Random Forest (RF). The CDF strategy allowed estimation of the significance of each peptide through permutations, providing a robust and sensitive ranking even with a small sample size. Complementarily, Random Forest was used to assess the classification ability of the peptides, and the ranking derived from the Gini index showed good agreement with the CDF results. The combination of both methods enabled the selection of peptides with consistent differential behavior, which were then grouped at the protein level according to reliability criteria: signal intensity (detectability of the peptides), internal consistency (multiple peptides from the same protein showing consistent differential behavior), and analytical robustness (experimental support through the detection of at least two significant peptides per protein, ensuring stability against technical variation). Additionally, cross-validation information with previously identified MALDI-TOF/TOF biomarkers was incorporated. A more detailed description of the two complementary approaches used for peptide ranking, RF and CDF analysis, is provided below.

- Cumulative Distribution Function-Based Analysis

For the identification and statistical significance assessment of peptides, we developed a nonparametric permutation-based approach that evaluates variable importance through cumulative distribution function (CDF) analysis [34]. This method quantifies the association between each peptide’s intensity profile and cancer status (class 1) versus control (class 0) by comparing the observed CDF pattern against what would be expected under the null hypothesis of no association.

For each peptide intensity variable P_i_ (where i = 1, 2, …, p), we consider the intensity values x_1_, x_2_, …, x_n_ across all samples and their corresponding class labels y_1_, y_2_, …, y_n_, where y_j_ = 1 indicates cancer and y_j_ = 0 indicates control. We first sort the observations in ascending order based on the peptide intensity values, resulting in ordered pairs (x_(1)_, y_(1)_), (x_(2)_, y_(2)_), …, (x_(n)_, y_(n)_), where x_(1)_ ≤ x_(2)_ ≤ … ≤ x_(n)_. The empirical CDF for cancer cases is calculated as shown in Equation (2):

(2) $${\mathrm{CDF}}_{\mathrm{real}}\left(j\right)=\frac{\sum_{k=1}^{j}y_{\left(k\right)}}{\sum_{k=1}^{n}y_{\left(k\right)}}\mathrm{for}\;j=1,2,\ldots ,n$$

To establish the null distribution under no association, we generate B = 1000 permutation surrogates by randomly shuffling the class labels while maintaining the peptide intensity ordering. For each permutation b, we compute the surrogate CDF (Equation (3)):

(3) $${\mathrm{CDF}}_{\mathrm{surrogate}\;(b)}^{b}(j)=\frac{\sum_{k=1}^{j}y_{\mathrm{perm}(k)}^{b}}{\sum_{k=1}^{n}y_{\mathrm{perm}(k)}^{b}}$$

The significance of each peptide is quantified using the S statistic, defined as the maximum standardized deviation between the real CDF and the permutation-based CDFs, as defined in Equation (4):

(4) $$S=\underset{j=1,\ldots ,n}{max}\frac{|{\mathrm{CDF}}_{\mathrm{real}}(j)-\mu_{\mathrm{surrogate}}(j)|}{\sigma_{\mathrm{surrogate}}(j)}$$

where µ_surrogate_(j) and σ_surrogate_(j) is the mean and standard deviation, respectively, of the surrogate CDFs at the j-th ordered data point.

Peptides were ranked according to the magnitude of their S statistic. Those with S ≥ 3 were classified as having high significance, values between 2 and 3 were considered moderately significant, and values between 1 and 2 were classified as low significance. Peptides with high S values exhibit intensity patterns where the cumulative distribution of cancer cases significantly deviates from what is expected by chance, suggesting their potential role as differential peptides between healthy and disease states.

- Random Forest-Based Analysis

To identify discriminative peptide features from the serum proteomic dataset, a supervised machine learning workflow based on a Random Forest (RF) classifier [39] combined with a Leave-One-Out Cross-Validation (LOOCV) scheme was implemented. This approach allows robust evaluation of predictive performance while providing a stable estimation of peptide importance across all samples. The RF classifier was configured with 100 decision trees in each LOOCV iteration. For a dataset with N samples, LOOCV constructs N independent RF models. In each iteration, one sample was withheld as the test instance while the remaining samples were used to train the RF model. The trained RF model produced a predicted class label and the probability of belonging to the cancer group for the left-out sample.

To quantify peptide relevance, the Mean Decrease Gini (MDG) metric was extracted from each RF model. MDG measures the cumulative reduction in node impurity attributed to splits involving a given peptide across all trees in the forest. The final importance score for each peptide was computed by averaging its Gini importance across all LOOCV iterations, ensuring a stable and robust ranking (Equation (5)):

(5) $$S=\underset{j=1,\ldots ,n}{max}\frac{|{\mathrm{CDF}}_{\mathrm{real}}(j)-\mu_{\mathrm{surrogate}}(j)|}{\sigma_{\mathrm{surrogate}}(j)}$$

Predictive performance of the classifier was evaluated using LOOCV predictions, computing accuracy, sensitivity, and specificity based on true and predicted class labels. Peptides were then ranked according to their averaged Gini importance scores, providing a robust assessment of their contribution to the discrimination between control and cancer group samples. Classifier performance was visualized using two complementary plots: a Receiver Operating Characteristic (ROC) curve with the corresponding Area Under the Curve (AUC), summarizing overall discriminative ability, and a patient-level probability plot, showing the predicted probabilities for each sample with respect to the cancer threshold and highlighting True Positives (TP), True Negatives (TN), False Positives (FP), and False Negatives (FN).

## 3. Results and Discussion

### 3.1. Quantitative Assessment of Serum Protein Spots by 2D-DIGE

**Figure 2:**
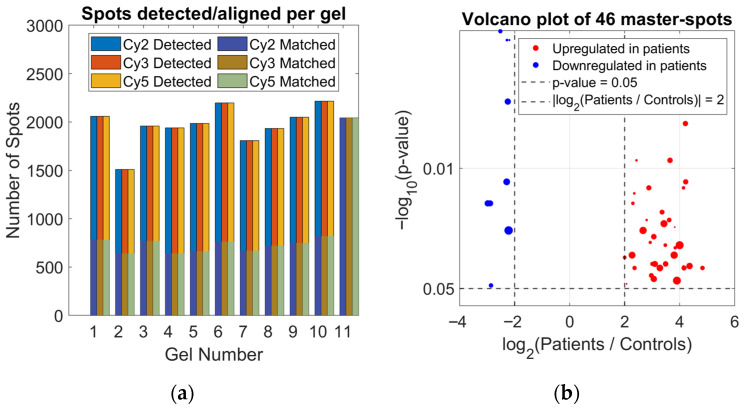
Integrative analysis of DIGE gels showing protein spot detection, differential expression, and reproducibility: (**a**) Distribution of detected and aligned spots across 33 gels for Cy2, Cy3, and Cy5 channels, highlighting the robustness of spot alignment across technical and biological replicates; (**b**) Volcano plot of 46 selected master-spots (present in ≥ 24 gels, *t*-test < 0.05, |log_2_(Average Ratio of Group1/Group2)| ≥ 2, corresponding to a two-fold change), highlighting upregulated proteins in red and downregulated proteins in blue, with marker size proportional to alignment quality (Match Quality) to indicate the reliability of each spot.

A total of 33 DIGE gels were analyzed, including three channels per gel: internal standard (Cy2), breast cancer patient samples (Group 1, Cy3), and healthy controls (Group 2, Cy5). The number of detected spots per gel ranged from 1509 to 2215, depending on the channel and the specific gel. After alignment with the Master Spot Map, between 614 and 820 spots were correctly aligned, providing a robust basis for quantitative and statistical analysis. This high alignment reproducibility across technical and biological replicates underscores the reliability of the dataset (Figure 2a).

To identify differentially expressed proteins, stringent selection criteria were applied, including the presence of the spot in at least 24 of 33 gels, *t*-test < 0.05, and |log_2_(Average Ratio of Group1/Group2)| ≥ 2. Using these filters, 46 master spots were selected for further analysis. Each master spot represents the same protein location across all gels, allowing direct comparison of fluorescence intensities between cancer patients and controls. The selected spots showed either increased abundance in patients (upregulated, e.g., Master No. 80, Av. Ratio = +4.22) or decreased abundance in patients (downregulated, e.g., Master No. 809, Av. Ratio = −2.52), reflecting complex modulation of serum protein expression associated with disease.

The significance and magnitude of these changes are summarized visually in a volcano plot, highlighting upregulated proteins in red and downregulated proteins in blue (Figure 2b). Point size is proportional to alignment quality (Match Quality), so larger points indicate spots with more reliable detection across gels. Dashed lines indicate the significance threshold (*p* = 0.05) and the fold-change threshold of |log_2_(Average Ratio of Group1/Group2)| = 2, which corresponds to a two-fold change and represents large differences in protein abundance. Positive log_2_ ratios indicate higher protein abundance in patients (upregulated, shown in red), whereas negative values indicate higher abundance in controls (downregulated, shown in blue).

**Figure 3:**
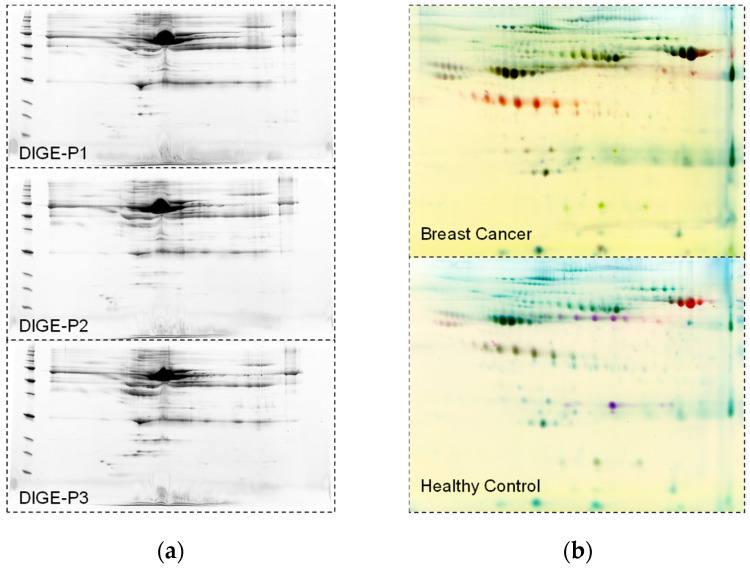
Representative 2-D DIGE analysis of serum proteins for quantitative assessment of patient and control samples: (**a**) Representative two-dimensional DIGE separations of serum proteins from three cancer patients, with the first dimension on an 11 cm pH 3–10 strip and the second dimension across an 8–16% range, showing individual protein spots detectable across channels for quantitative analysis; (**b**) Representative 2-D DIGE gels show a breast cancer sample in the top panel and a healthy control sample in the bottom panel, with blue (Cy2) indicating the internal standard, green (Cy3) the patient sample, and red (Cy5) the healthy control.

To further illustrate the quantitative assessment of serum protein spots, Figure 3 presents representative 2-D DIGE analyses. Figure 3a shows the separations of serum proteins from three breast cancer patients, with the first dimension on an 11 cm pH 3–10 strip and the second dimension across an 8–16% gradient, highlighting individual protein spots detectable across channels for quantitative analysis. Figure 3b displays representative 2-D DIGE gels of a breast cancer patient sample (top panel) and a healthy control sample (bottom panel), with blue (Cy2) indicating the internal standard, green (Cy3) the patient sample, and red (Cy5) the healthy control, providing a visual confirmation of spot distribution and channel reproducibility across the dataset.

### 3.2. Biomarker Identification Using MALDI-TOF

The analysis of spots obtained by 2D DIGE gel and MALDI TOF/TOF generated peptide mass fingerprints (PMFs), which were compared against the NCBInr and Swiss Prot databases for protein identification. This combined strategy provided broad coverage and high annotation confidence, as Swiss Prot offers curated and validated sequences, while NCBInr extends the search to a larger repertoire of known proteins. Proteins consistently identified in both databases were considered highly reliable, whereas discrepancies were primarily attributed to differences in sequence completeness, database curation, or nomenclature variations. In total, 48 spots were analyzed, of which 8 proteins were selected as potential breast cancer biomarkers in serum from African American women (Table 1).

Swiss-Prot Hit Mass and Hit Score reflect the match between the experimental peptide fingerprint and the theoretical database sequence, with higher values indicating greater confidence in the identification. For example, Alpha-1-antitrypsin (A1AT) shows a Hit Score of 1076, indicating a very strong match, whereas Complement C3 (CO3), with a Hit Score of 256, represents a moderate but significant identification. Parameters obtained from NCBInr, including ProteinMW (theoretical molecular weight), ProteinPI (isoelectric point), ProteinScore (confidence statistic), and PepCount (number of identified peptides), allow validation of structural consistency and sequence coverage. Proteins with high ProteinScore and PepCount, such as Ceruloplasmin (CERU; 400, 33) or Alpha-2-macroglobulin (A2M; 367, 42), represent robust and reliable identifications. In contrast, proteins with lower peptide coverage, such as Alpha-2-HS-glycoprotein (FETUA; PepCount 7), although identified, have less structural support and should be interpreted with caution. The AV Ratio values presented in Table 1, obtained from 2D DIGE analysis, reflect relative changes in protein abundance between patients and controls, indicating overexpression (positive values) or underexpression (negative values). For instance, Alpha-1B-glycoprotein (A1BG) has an AV Ratio of 4.36, indicating a marked increase in patients, whereas Alpha-2-HS-glycoprotein (FETUA) shows an AV Ratio of −2.86, indicating a significant decrease. This parameter integrates both the magnitude and direction of regulation, enabling prioritization of differentially expressed proteins with potential biological or clinical relevance.

**Figure 4:**
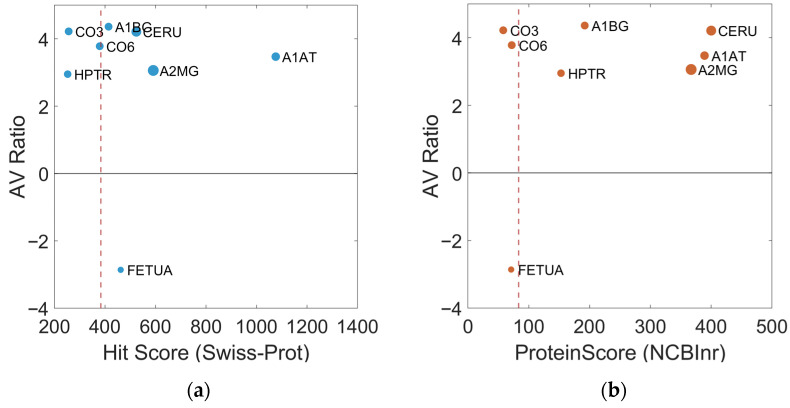
Dual-parameter scatter plot showing identification confidence: (**a**) X-axis: Swiss-Prot Hit Score, and (**b**) NCBInr ProteinScore versus expression changes (Y: AV Ratio). Point size represents peptide coverage (PepCount). Dotted lines indicate confidence thresholds (first third of the distribution: (**a**) Hit Score = 384; (**b**) ProteinScore = 83) which distinguish high- from moderate-confidence proteins.

To comprehensively assess both identification reliability and biological relevance, a dual-parameter scatter plot analysis was performed (Figure 4) integrating database confidence metrics with quantitative expression changes. This visualization displays identification confidence scores (X-axis: Swiss-Prot Hit Score in Figure 4a; NCBInr ProteinScore in Figure 4b) against expression changes (Y-axis: AV Ratio), with point sizes proportional to peptide coverage (PepCount). Confidence thresholds, defined at the first third of each score distribution, distinguish high-confidence from moderate-confidence proteins. This integrated approach revealed distinctive biomarker profiles: proteins such as A1AT exhibited exceptional identification confidence (Hit Score: 1076) together with marked overexpression (AV Ratio: 3.47), identifying them as high-priority candidates. In contrast, CO3 showed the largest expression change (AV Ratio: 4.22) despite moderate confidence scores, highlighting its potential biological relevance while indicating the need for further validation. The analysis also distinguished proteins with consistently high confidence across both databases (CERU, A2MG) from those with database-specific confidence patterns, enabling stratified prioritization based on both technical reliability and biological relevance.

The proteins listed in Table 1, as described in UniProtKB/Swiss-Prot, the manually reviewed and annotated protein database maintained by the UniProt Consortium [40], exhibit diverse physiological roles. Ceruloplasmin (CERU) is a copper-binding glycoprotein with ferroxidase activity. Alpha-2-macroglobulin (A2MG) inhibits all classes of proteinases via a trapping mechanism, while alpha-1-antitrypsin (A1AT) primarily inhibits elastase and moderately binds plasmin and thrombin. Complement components C3 and C6 (CO3 and CO6) participate in the complement system, with C6 forming part of the membrane attack complex. Alpha-1B glycoprotein (A1BG) is a plasma glycoprotein of unknown function similar to immunoglobulin variable regions. Alpha-2-HS-Glycoprotein (FETUA) contains two cystatin-like domains. The haptoglobin-related protein (HPTR) is associated with apolipoprotein L-I-containing high-density lipoproteins. Finally, the constant regions of immunoglobulins, IGHG1, IGHA1, and IGKC are produced by B lymphocytes as secreted or membrane-bound glycoproteins mediating humoral immune responses.

Given their diverse physiological functions and roles in processes such as complement regulation, protease inhibition, and immune response, these proteins also exhibit significant expression changes in the serum of breast cancer patients, suggesting their potential relevance as biomarkers. Among the candidates analyzed, Alpha 1 antitrypsin (A1AT) emerged as particularly noteworthy for its high expression (AV Ratio = 3.47) and highest identification scores, establishing it as a priority marker. Previous studies have documented that inhibition of α1-antitrypsin (α1-AT) in triple-negative carcinoma cells reduces viability, migration, and invasion by modulating the PI3K/Akt/mTOR pathway and regulating metastatic factors such as E-cadherin, TIMP-2, MTA1, and MMP2, supporting its biological relevance and potential as a therapeutic target [41].

Similarly, C3 and C6, components of the complement system, showed significant serum elevations (AV Ratios 4.22 and 3.78, respectively). While C3a contributes to the activation of the tumor microenvironment and promotes metastatic progression through fibroblast activation, production of pro-tumor cytokines such as TGF-β, and recruitment of immunosuppressive and prometastatic neutrophils [42]. In addition, C6 is an essential component of the membrane attack complex (C5b–9), which may be involved in modulating complement-mediated cytotoxicity and has recently been proposed as an early serum biomarker of breast cancer [43].

Ceruloplasmin (CERU) showed overexpression in serum (AV Ratio = 4.21) in our patients, in agreement with previous reports describing significantly elevated serum levels in advanced or metastatic tumors. Although CERU is an acute-phase reactant, it has been noted that this does not diminish its diagnostic utility, and its increase could reflect both tumor burden and systemic inflammatory response [44].

Also, Alpha 1B-glycoprotein (A1BG) also showed expression changes in the serum of breast cancer patients (AV Ratio = 4.36), highlighting its potential relevance as a biomarker. Its signal was primarily detected in inflammatory cells present in the tumor and lymph nodes, and low expression was associated with worse metastasis-free survival, suggesting a role in modulating the tumor immune response. Although its precise function is not yet fully defined, these findings support its potential utility for monitoring the interaction between tumor and immune microenvironment [45].

Alpha 2 macroglobulin (A2MG) further underscores the importance of protease inhibitors in tumor regulation, appearing with an AV Ratio = 3.06. This protein functions as a broad-spectrum protease inhibitor with multifaceted roles in regulating the tumor microenvironment. It binds cytokines, growth factors, and misfolded proteins, modulating proteolytic activity and cancer-related cellular signaling. Recent studies in breast cancer have shown that tissue levels of A2MG may be up- or downregulated depending on the tumor context, and its expression correlates with cellular aggressiveness [46]. In our context, elevated serum A2MG could reflect a response to extracellular matrix degradation or protease activation, supporting its potential relevance as a functional biomarker and candidate for clinical monitoring.

The haptoglobin-related protein precursor (HPTR) exhibited an AV ratio of 2.95 in our analysis. Kuhajda et al. [47] reported that HPTR acts as a potential predictor of recurrence in early-stage breast carcinoma and is independently associated with a higher risk of recurrence, even when accounting for other prognostic factors such as tumor size or hormone receptor status. Thus, elevated serum HPTR could indicate a tumor microenvironment with increased aggressiveness or recurrence risk, supporting its consideration as a potential progression biomarker in breast cancer.

Finally, Alpha 2 HS glycoprotein precursor (FETUA) provides additional insight into tumor–host interactions, presenting an AV Ratio = −2.86 in our study. This plasma glycoprotein has multiple physiological functions, from regulation of calcification to modulation of tumor growth signaling, and in breast cancer it has been documented to act as a cell adhesion factor through interaction with annexin family proteins, contributing to proliferation and potential invasion of mammary tumor cell lines [48]. Its serum elevation could reflect both increased tumor activity and systemic changes in host response, positioning it as a promising marker for detection or monitoring in breast cancer.

### 3.3. Biomarker Identification Using Shotgun LC–MS/MS

The shotgun LC–MS/MS analysis generated a dataset that integrates, for each detected peptide, fundamental analytical parameters such as molecular mass, retention time, and their standard deviations, as well as the detection frequency across samples. This allows for the assessment of instrumental stability, the coverage of each signal, and variability between subjects, including the distribution of missing values, which are typically associated with low-abundance or inconsistently detected signals. The dataset also includes comparative metrics such as Av. Diff. (2Log) and Av. Ratio (Lin), along with *t*-test *p*-values, which facilitate the identification of quantitative differences between the control and breast cancer groups.

Since average-based parameters can be misleading in the presence of high variability or incomplete data, stricter filtering and imputation were applied, and methods that analyze complete patterns rather than means, such as machine learning algorithms or non-parametric statistics, are considered for biomarker identification. Therefore, the following section describes the procedures and results of preprocessing and imputation applied to the LC–MS/MS dataset, which form the basis for subsequent analyses aimed at biomarker identification using approaches such as Random Forest (Gini index) and cumulative distribution function (CDF)-based analysis.

#### 3.3.1. LC–MS/MS Data Preprocessing and Imputation Results

The initial dataset consisted of 286 peptides measured across 12 samples, corresponding to six controls and six breast cancer samples, totaling 3432 observations. Of these, 651 (19.0%) were missing values, mainly associated with low intensities below the detection limit. To address these missing data, the MinProb method [37] was applied, using a downshift of 1.8 in the log_2_ scale and a width of 0.3. This procedure ensures that the imputed values reflect low intensities that were originally undetected, while preserving the overall shape of the observed data distribution.

**Figure 5:**
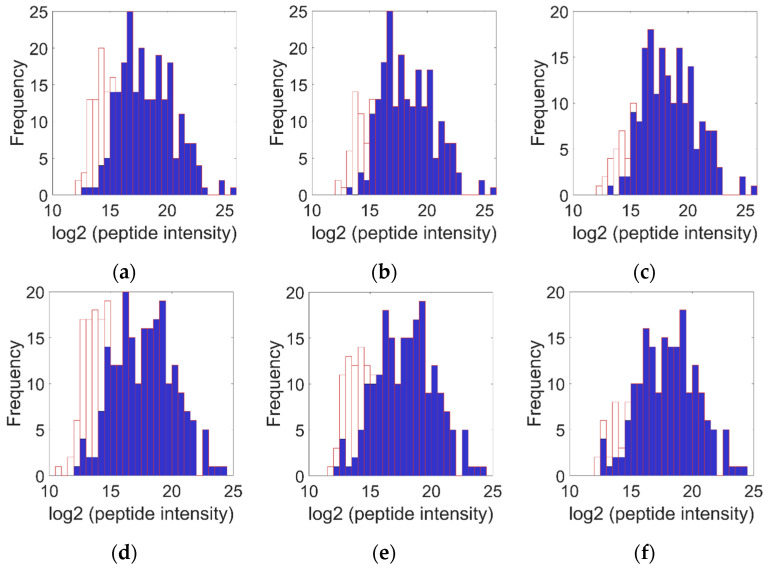
Peptide intensity distributions after imputation at different minimum observation thresholds (minSamples): Panels (**a**–**c**) show a representative control sample, and panels (**d**–**f**) a representative breast cancer sample. Within each group, panels correspond to minSamples = 2 (left), 3 (middle), and 4 (right). Increasing minSamples removes peptides with few observations and improves data reliability. Blue (filled) histograms show the distribution of observed peptide intensities, whereas open (unfilled) histograms show the distribution of imputed peptide intensities.

To ensure the reliability of the retained data, filtering based on minimum observation coverage per peptide (minSamples) was applied, evaluating minSamples values of 2, 3, and 4. Figure 5 shows the resulting intensity histograms. Panels a–c present a representative example of the control sample, while panels d–f show an example of the breast cancer sample. Within each group (a–c and d–f), panels are presented in the same order: minSamples = 2 in the first panel, minSamples = 3 in the second, and minSamples = 4 in the third. This organization allows for comparison of how increasing the minimum observation coverage threshold per peptide affects the intensity distribution and the proportion of imputed versus observed values.

In the full set of observations, with minSamples = 2, no peptides were removed, and all imputed (651, 19.0%) and observed (2781, 81.0%) values were retained. However, the distribution of imputed intensities (unfilled histograms) exhibited marked bimodality, particularly in the control samples, indicating an artificial bias due to the inclusion of peptides with very low observation coverage. It is worth noting that, although standard procedures with minSamples = 2 typically apply a Student *t*-test (and associated *p*-values) automatically, in our data the intensity histograms (Figure 5a,d) show a clear bimodal distribution, invalidating the t-test assumption of normality. This justifies the use of stricter filtering and alternative methods that analyze patterns rather than means, such as machine learning or non-parametric statistics.

Increasing the criterion to minSamples = 3 resulted in the removal of 39 peptides (13.6%) and 468 values, of which 210 were observed (7.6% of the total observed) and 258 imputed (39.6% of the total imputed). After this intermediate filtering, 247 peptides remained, reducing the bimodality observed with minSamples = 2, but still retaining some peptides with insufficient coverage that may bias the intensity distribution. Although this improves consistency with direct measurements, this threshold is not sufficient to ensure maximum data reliability.

Finally, with minSamples = 4, 88 peptides (30.8%) and 1056 values were removed, including 565 observed (20.3% of the total observed) and 491 imputed (75.4% of the total imputed). After this strict filtering, 198 peptides remained, ensuring that most had sufficient coverage, eliminating bimodality, and primarily reflecting reliable measurements. In this set, 2216 observed values (93.3% of the total observed) and 160 imputed values (6.7% of the total imputed) were retained. Subsequently, entries corresponding to identical peptides were collapsed by calculating the mean of their intensities, reducing the final dataset to 163 unique peptides, which served as the basis for biomarker identification using Cumulative Distribution Function and Random Forest analysis.

#### 3.3.2. Biomarker Analysis Using Cumulative Distribution Function and Random Forest

Biomarker analysis based on the cumulative distribution function (CDF) enabled precise characterization of the distributional behavior of the 163 peptides evaluated, through estimation of the statistic S derived from the maximum deviation between the observed CDF and the permuted null distributions (Equation (4)). Here, S represents the deviation in units of standard deviation (SD) from the null distribution, i.e., S = 1, 2, 3 correspond to 1 SD, 2 SD, and 3 SD, respectively. Classification of the S values revealed a marked concentration in the intermediate intervals, with 48.5% of the peptides falling within the moderate significance range (2 ≤ S < 3) and 49.7% in the low significance range (1 ≤ S < 2). Only 1.2% reached S ≥ 3, represented by two peptides with highly differential behavior, while a mere 0.6% displayed non-significant values (S < 1). In absolute terms, 81 peptides exceeded the primary threshold of S > 2, and almost all (162/163) exhibited S > 1. This indicates that the majority of peptides show some degree of differentiation between cases and controls, demonstrating that the influence of clinical status is consistently reflected across the dataset. The internal distribution was likewise homogeneous, with 79 peptides in the interval 2 ≤ S < 3 and 81 peptides in 1 ≤ S < 2. This profile suggests that the CDF-based approach captures relevant distributional alterations with high sensitivity and provides a robust foundation for progressing toward protein-level analysis.

**Figure 6:**
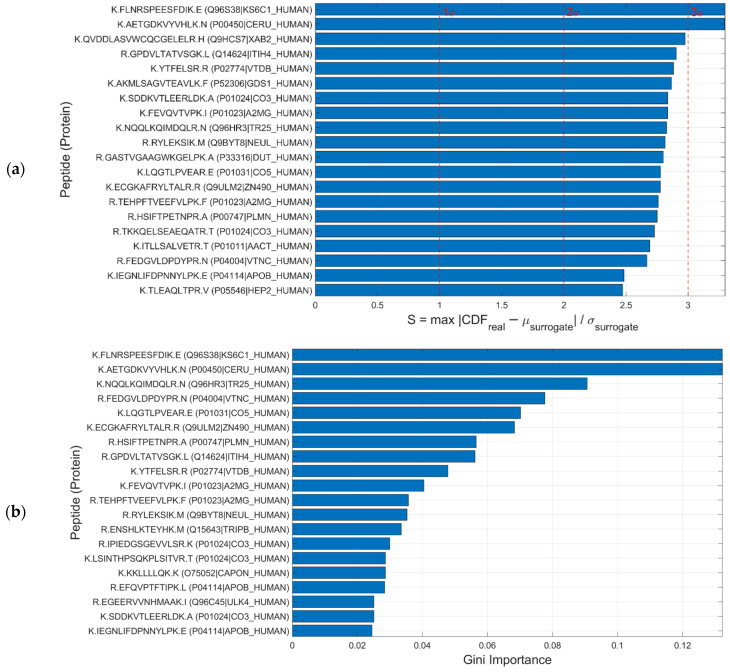
Comparison of (**a**) CDF S values and (**b**) Random Forest (Gini) scores for a representative set of peptides. In panel (**a**), the vertical lines indicate S values of 1, 2, and 3, corresponding to thresholds of increasing significance expressed as multiples of the standard deviation (σ) from the null distribution in the cumulative distribution function (CDF) analysis. For clarity, only the top 20 peptides (ranked among the 163 analyzed) are shown; comprehensive protein-level rankings would require additional criteria, as discussed throughout this section.

Complementarily, peptide classification performance was evaluated using a Random Forest (RF) model with a Leave-One-Out Cross-Validation (LOOCV) scheme. Despite the limited sample size (12 samples in total, 6 per group), the variable ranking based on Mean Decrease Gini (MDG) showed clear concordance with the CDF results: peptides with high significance (S ≥ 3) corresponded closely, while those with intermediate MDG relevance aligned with the moderate significance category in CDF (2 ≤ S < 3) (Figure 6). This agreement underscores the robustness of the findings, indicating that both approaches yield consistent and mutually reinforcing insights. Importantly, these results apply at the peptide level and do not represent a global ranking of all proteins to which they belong, which would require additional criteria, as discussed throughout Section 3. For illustrative purposes, only the top 20 peptides of the 163 analyzed are shown.

**Figure 7:**
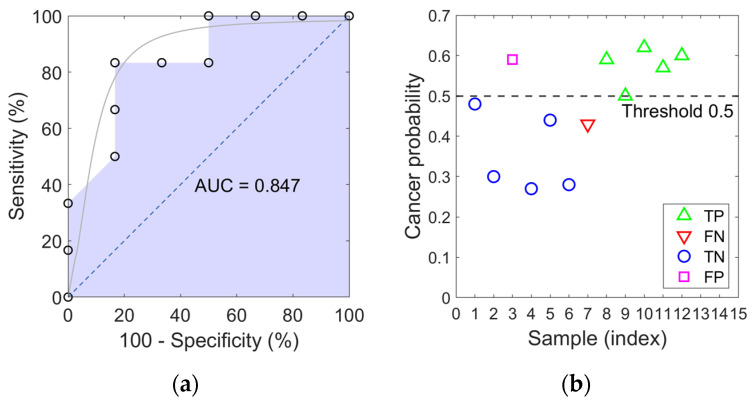
Classifier performance evaluation: (**a**) Receiver Operating Characteristic (ROC) curve with Area Under the Curve (AUC) indicating model discriminative ability, and (**b**) patient-level predicted probabilities showing True Positives (TP), True Negatives (TN), False Positives (FP), and False Negatives (FN).

The performance of the classifier was illustrated through two complementary visualizations. The Receiver Operating Characteristic (ROC) curve summarized the relationship between sensitivity (%) and 100-specificity (%) across the thresholds evaluated during the LOOCV procedure, exhibiting an upward-trending trajectory characteristic of a model with appreciable discriminative ability. The resulting Area Under the Curve (AUC = 0.847) represents the area beneath the shaded ROC plot, providing a visual summary of the model discriminative ability (Figure 7a). The curve exhibits discrete rises, reflecting the limited number of predictions due to the small sample size. To aid visual interpretation, a light gray sigmoidal interpolation was overlaid to highlight the overall trend; this line is purely illustrative and carries no statistical meaning. The diagonal line represents the performance of a random classifier, serving as a reference: points above this line indicate better-than-random discrimination between cases and controls. Complementarily, a patient-level probability plot displayed the predicted probabilities for each sample, enabling the visual identification of True Positives (TP), True Negatives (TN), False Positives (FP), and False Negatives (FN). This representation provided an intuitive view of how confidently the model assigned each observation to its corresponding class (Figure 7b).

In this study, CDF analysis was employed as a central tool throughout the downstream processing to identify proteins of interest, providing direct statistical significance, which allows a rigorous assessment of which peptides exhibit relevant associations with disease status. This nonparametric, permutation-based approach evaluates the relevance of each peptide independently, without assuming specific distributions or relying on correlations between variables, offering robust and stable importance measures even with a limited number of samples. The observed concordance with Random Forest results supports the consistency of the identified relevance patterns, demonstrating that both approaches provide complementary information. While the conventional RF approach can offer additional advantages in studies with larger cohorts, in this work it serves to illustrate the relative contribution of the peptides and provide interpretative insights into their behavior. In contrast, the proposed CDF-based approach provides direct statistical significance for each peptide, offering a robust and novel strategy for evaluating proteomic data even with limited sample sizes.

Based on the peptide-level results, the data were integrated to assess protein-level information, organized into columns reflecting signal magnitude, internal consistency, and analytical robustness. Documented variables include the UniProt identifier of the protein precursor (Protein_ID), the number of peptides exceeding the significance threshold (n_peptides_S, ≥1; in bold: ≥2), the total number of detected peptides (n_peptides_total), the proportion of significant peptides (ratio_peptides_S), the maximum S value among the peptides (S_max_peptides), and compliance with the minimum experimental support criterion, defined as the detection of at least two peptides. In addition, cross-validation labels of previously identified biomarkers using MALDI-TOF/TOF (Section 3.2: Biomarker Identification using MALDI-TOF) are included to evaluate the external consistency of the candidates. Table 2 summarizes these data for the first-level evaluation, showing all proteins with at least one significant peptide (n_peptides_S ≥ 1, less stringent criterion) and highlighting in bold those with two or more significant peptides (n_peptides_S ≥ 2, more stringent criterion). Among the latter, 13 proteins are identified with strong evidence: CERU, A2MG, CO3, VTDB, HEMO, APOB, APOA4, CFAH, CO4A, AACT, K1C10, ITIH2, and ITIH4.

Table 3 lists the individual peptides corresponding to the 13 proteins classified as strong-evidence candidates (highlighted in bold in Table 2). These proteins meet strict reliability criteria, being supported by at least two significant peptides (S > 2) and by more than two experimentally detected peptides with quantified intensities. The table includes flanking residues and the full sequence context for each fragment, enabling assessment of protein-level signal consistency across constituent peptides and identification of the peptide regions driving case–control differentiation. This information provides a solid basis for targeted mass spectrometry quantification and subsequent biomarker validation, as well as guidance for further analyses of specific proteins and peptides in clinical or experimental studies.

Among the identified serum biomarkers, ceruloplasmin (CERU) exhibited a robust signal across both approaches. In the LC–MS/MS analysis processed with CDF, two significant peptides were detected with a maximum S value (S_max) of 3.29, whereas MALDI-TOF/TOF reported 33 identified peptides, with an AV Ratio of 4.21 and a Hit Score of 524, confirming both overexpression in patients and the structural reliability of the protein. Ceruloplasmin is a copper-binding glycoprotein with ferroxidase activity, and its serum elevation has been previously associated with advanced or metastatic tumors, reflecting systemic inflammatory response and tumor burden [44].

Alpha-2-macroglobulin (A2MG) also showed concordance between both methods. In the CDF analysis, six significant peptides were detected with an S_max of 2.83, while MALDI-TOF/TOF identified 42 peptides with an AV Ratio of 3.06 and a Hit Score of 591. This protein functions as a broad-spectrum protease inhibitor and modulates cytokine and growth factor activity within the tumor microenvironment. Its serum elevation may reflect the body response to extracellular matrix degradation or proteolytic activation, establishing it as a functionally relevant clinical biomarker [46].

Complement C3 (CO3) was identified with nine significant peptides and an S_max of 2.83 in the LC–MS/MS CDF analysis, showing an AV Ratio of 4.22 in MALDI-TOF/TOF, indicating marked overexpression in patients. Although the Hit Score was moderate (256), the biological relevance of C3 is evident, as it actively participates in tumor microenvironment activation, promoting the production of pro-tumor cytokines, fibroblast activation, and the migration of immunosuppressive and pro-metastatic neutrophils [42].

In the case of alpha-1-antichymotrypsin (AACT), the LC–MS/MS analysis processed with CDF identified three significant peptides out of four, with a maximum S value (S_max) of 2.6915, indicating clear differentiation between cases and controls. Although AACT was not detected in the MALDI-TOF/TOF analysis in this study, its biological relevance is well supported in the literature. This protein is a chymotrypsin-type serine protease inhibitor and is functionally related to alpha-1-antitrypsin (A1AT). Zhao et al. [41] have shown that modulation of these proteins can affect the viability, migration, and invasion of triple-negative carcinoma cells through regulation of the PI3K/Akt/mTOR pathway and metastasis-associated factors such as E-cadherin, TIMP-2, MTA1, and MMP2, further supporting the potential of AACT as a functional serum biomarker.

In Table 2, VTDB (Vitamin D-binding protein) and HEMO (Hemopexin) exhibit a serum differentiation pattern that is noteworthy from the perspective of complementary biomarker potential. In the LC–MS/MS analysis processed with CDF, VTDB showed 2 significant peptides out of 4, with a maximum S value (S_max) of 2.8825, while Hemopexin also had 2 out of 3 peptides with an S_max of 2.3691, indicating a clear separation between cases and controls. Although the role of VTDB in breast cancer is not yet fully characterized, VTDB (GC) has been identified among HDL-associated proteins with differential abundance according to tumor molecular subtype, with differences observed between triple-negative and luminal or HER2 subtypes [49]. Hemopexin, on the other hand, has been proposed as a regulator of tumor growth by sequestering free heme, counteracting its pro-oxidative effects; this function has been discussed in the context of cancer in general [50].

Furthermore, additional proteins listed in Table 2 provide relevant information on metabolic and signaling processes. For example, APOB (Apolipoprotein B-100) displayed between 2 and 6 significant peptides (S_max between 2.3306 and 2.3616), potentially reflecting alterations in lipid metabolism; elevated APOB levels have been associated with increased recurrence in breast cancer [51]. Similarly, APOA4 (Apolipoprotein A-IV) has been linked in epidemiological studies to a lower cancer risk when serum levels are higher [52].

Regarding proteins involved in complement regulation, CFAH (Complement Factor H) showed 3/3 significant peptides with an S_max of 2.363, whereas CO4A (Complement C4A) presented 6/11 peptides with an S_max of 2.3616. Factor H regulates the alternative complement pathway by controlling C3 activation and protecting host cells from excessive activation, while C4A is a key component of the classical and lectin pathways, participating in opsonization and immune complex clearance [40]. Although a direct link with breast cancer has not been established, the magnitude and consistency of the S values suggest potential alterations in complement activity in serum, warranting further functional investigation in future studies.

Furthermore, the inter-alpha-trypsin inhibitor heavy chains ITIH2 and ITIH4, together with keratin K1C10, displayed 2–3 significant peptides with S_max values ranging from 2.2836 to 2.905. For ITIH2, although no serum-specific studies in breast cancer have been reported, its biological role is well described: it belongs to the inter-α-inhibitor family and participates in extracellular matrix stabilization during inflammatory processes [40]. ITIH4 has been quantified in the serum of breast cancer patients, with several peptide fragments (e.g., residues 658–687) showing higher levels in patients compared to controls, and several of these fragments decreased after surgery [53]. Regarding K1C10, this keratin has been detected by immunohistochemistry in approximately 16% of primary breast carcinomas, and its high expression has been associated with poorer prognosis in breast cancer patients [54].

In addition to the prioritized candidates (n_peptides_S ≥ 2, more stringent criterion), Table 2 shows all proteins with at least one significant peptide (n_peptides_S ≥ 1, less stringent criterion). Proteins with a single significant peptide are included under the less stringent criterion, and their functions are interpreted based on UniProtKB annotations [40]. ANT3 (Antithrombin-III) and ANGT (Angiotensinogen) each displayed one significant peptide, with S_max values of 2.3487 and 2.3621, respectively, suggesting intermediate differentiation between cases and controls. ANT3 inhibits thrombin and regulates coagulation, while ANGT is the precursor of angiotensins and participates in blood pressure regulation and renin–angiotensin system homeostasis. CO5 (Complement C5), RETBP (Retinol-binding protein 4), and VTNC (Vitronectin) also each had one significant peptide, with S_max values ranging from 2.337 to 2.7788. CO5 is a key component of the complement system contributing to immune activation; RETBP transports retinol (vitamin A) in the blood; and VTNC participates in cell adhesion and extracellular matrix stabilization, functions that may reflect modulation of the tumor microenvironment. Other proteins, HEP2 (Heparin cofactor II), CLUS (Clusterin), ITIH1 (Inter-alpha-trypsin inhibitor heavy chain H1), and ALS (Insulin-like growth factor–binding protein complex acid labile subunit), also displayed one significant peptide each, with S_max values between 2.0629 and 2.4736. HEP2 inhibits thrombin; CLUS protects cells against stress and regulates apoptosis; ITIH1 contributes to extracellular matrix stability; and ALS regulates the bioavailability of insulin-like growth factors, reflecting possible changes in signaling within the tumor microenvironment. Although each of these proteins has only a single significant peptide, their S values and biological functions suggest they may reflect relevant modulations in the tumor microenvironment and warrant further investigation in future studies.

Overall, these results demonstrate that the LC–MS/MS approach processed with CDF enables the identification of robust and quantifiable proteomic alterations in serum, based on objective metrics of significance and differentiation (S_max, n_peptides_S, ratio_peptides_S). Among the 13 proteins with strong evidence, CERU, A2MG, and CO3 stand out as prioritized candidates validated by MALDI-TOF/TOF, confirming their overexpression and structural reliability. The remaining proteins in the panel; VTDB, HEMO, AACT, APOB, APOA4, CFAH, CO4A, K1C10, ITIH2, and ITIH4, provide complementary information, as discussed above and supported by previous studies: VTDB and HEMO reflect differences according to tumor subtypes and oxidative stress; AACT modulates viability and invasion in triple-negative breast cancer cells; APOB and APOA4 have been associated with risk and recurrence of breast cancer; ITIH4 exhibits elevated serum fragments that decrease after surgery; and K1C10 has been detected in primary carcinomas with poorer prognosis. Within the context of an analysis aimed at characterizing serum protein signatures associated with breast cancer in treatment-naïve African American women, these results are particularly relevant. Although some proteins lack cross-validation in MALDI-TOF/TOF, the detection of significant peptides highlights the sensitivity of the CDF-processed LC–MS/MS approach in capturing functional changes in tumor microenvironment remodeling, immune regulation, lipid metabolism, and extracellular matrix stability, providing a solid foundation for subsequent functional validation studies and clinical exploration of this panel as serum biomarkers of breast cancer in African American women.

## 4. Conclusions

This study implemented an integrated strategy for the identification of serum biomarkers of breast cancer in African American women. Quantitative proteomics using 2D-DIGE, reliable protein identification by MALDI-TOF/TOF, and peptide-level discrimination by LC–MS/MS, combined with multivariate analyses using CDF and Random Forest, enabled the construction of a consistent and reproducible proteomic sensor platform capable of distinguishing breast cancer patients from healthy controls. 2D-DIGE enabled the generation of a consistent and reproducible proteomic map, detecting 46 gel spots with significant differential expression across replicates. Proteins from the analyzed spots were identified by MALDI-TOF/TOF and matched against curated databases, highlighting serum biomarkers such as Ceruloplasmin (CERU), Alpha-2-Macroglobulin (A2MG), complement components C3 and C6 (CO3 and CO6), Alpha-1-Antitrypsin (A1AT), Alpha-1B-Glycoprotein (A1BG), Alpha-2-HS-Glycoprotein (FETUA), and Haptoglobin-related Protein (HPTR). The biological roles of these proteins are consistent with prior literature: CERU is linked to cancer-associated inflammation and metastatic progression, A2MG regulates protease activity within the tumor microenvironment, CO3 and CO6 reflect complement activation during tumor progression, and A1AT contributes to modulation of tumor cell survival and invasion. LC–MS/MS analysis, after imputing missing values to reflect low-intensity signals, generated 163 unique differentiating peptides. These peptides were evaluated using the Cumulative Distribution Function (CDF), a non-parametric method that measures each peptide deviation from a null distribution generated by permutations, providing statistical significance even in small cohorts. Random Forest analysis, via Mean Decrease Gini, showed clear correspondence with CDF results in peptide importance, yielding an AUC of 0.847. Integrating all platforms and criteria, 13 differentiating serum proteins were prioritized: CERU, A2MG, CO3, VTDB, HEMO, APOB, APOA4, CFAH, CO4A, AACT, K1C10, ITIH2, and ITIH4. Although only CERU, A2MG, and CO3 were consistently identified by both DIGE–MALDI-TOF/TOF and LC–MS/MS, this partial overlap highlights the complementary detection capabilities of the two approaches. DIGE–MALDI-TOF/TOF preferentially identifies abundant, well-resolved serum proteins, whereas LC–MS/MS enables the detection of additional lower-abundance proteins not captured in 2D gels. This combined strategy supports a robust and comprehensive evaluation of serum proteins for breast cancer biomarkers. These biomarkers reflect key processes in breast cancer pathophysiology, including complement regulation, protease inhibition, tumor microenvironment remodeling, lipid metabolism, and immunomodulation. They also play a role in regulating cell proliferation, differentiation, and apoptosis, providing a solid basis for their potential use as serum markers in early detection and monitoring. It is important to note that this study focuses on African American women, a historically underrepresented group in breast cancer biomarker research, making these findings particularly relevant for this population, although validation in larger cohorts would help strengthen their clinical applicability.

## Figures and Tables

**Table 1:** Candidate Serum Biomarkers for Breast Cancer: Identification and Quantitative Metrics from 2D DIGE and MALDI TOF/TOF. Documented variables include the UniProt identifier of the protein precursor (Protein_ID).

Protein_ID	Hit Mass (Swiss-Prot)	Hit Score (Swiss-Prot)	ProteinMW (NCBInr)	Protein PI (NCBInr)	ProteinScore (NCBInr)	PepCount (NCBInr)	AV Ratio
P00450|CERU_HUMAN	122,983	524	115,398.4	5.43	400	33	4.21
P01023|A2MG_HUMAN	164,600	591	163,174.9	6	367	42	3.06
P01024|CO3_HUMAN	188,585	256	187,029.9	6.02	58	17	4.22
P01009|A1AT_HUMAN	46,878	1076	44,222.7	5.37	389	22	3.47
P13671|CO6_HUMAN	108,425	379	104,776	6.31	72	17	3.78
P04217|A1BG_HUMAN	54,809	414	51,908.4	5.65	192	17	4.36
P02765|FETUA_HUMAN	40,098	462	39,299.7	5.43	71	7	−2.86
P00739|HPTR_HUMAN	39,496	252	38,786.6	6.67	153	15	2.95

**Table 2:** Summary of peptide detection metrics per protein at the first level of evaluation. Documented variables include the UniProt identifier of the protein precursor (Protein_ID), the number of peptides exceeding the significance threshold (n_peptides_S ≥ 1; in bold: ≥2), the total number of detected peptides (n_peptides_total), the proportion of significant peptides (ratio_peptides_S), and the maximum S value among the associated peptides (S_max_peptides). The analysis considers a minimum experimental support criterion, defined as the detection of at least two peptides.

Protein_ID	n_peptides_S	n_peptides_total	ratio_peptides_S	S_max_peptides
**P00450|CERU_HUMAN**	**2**	**8**	**25**	**3.2944**
P01008|ANT3_HUMAN	1	3	33.333	2.3487
**P01011|AACT_HUMAN**	**3**	**4**	**75**	**2.6915**
P01019|ANGT_HUMAN	1	3	33.333	2.3621
**P01023|A2MG_HUMAN**	**6**	**8**	**75**	**2.8357**
**P01024|CO3_HUMAN**	**9**	**16**	**56.25**	**2.8358**
P01031|CO5_HUMAN	1	2	50	2.7788
P02753|RETBP_HUMAN	1	2	50	2.337
**P02774|VTDB_HUMAN**	**2**	**4**	**50**	**2.8825**
**P02790|HEMO_HUMAN**	**2**	**3**	**66.667**	**2.3691**
P04004|VTNC_HUMAN	1	2	50	2.6691
**P04114|APOB_HUMAN**	**4**	**5**	**80**	**2.4843**
P05546|HEP2_HUMAN	1	2	50	2.4736
**P06727|APOA4_HUMAN**	**2**	**2**	**100**	**2.3306**
**P08603|CFAH_HUMAN**	**3**	**3**	**100**	**2.363**
**P0C0L4|CO4A_HUMAN**	**6**	**11**	**54.545**	**2.3616**
P10909|CLUS_HUMAN	1	3	33.333	2.4345
**P13645|K1C10_HUMAN**	**3**	**4**	**75**	**2.3712**
**P19823|ITIH2_HUMAN**	**2**	**4**	**50**	**2.2836**
P19827|ITIH1_HUMAN	1	3	33.333	2.0629
P35858|ALS_HUMAN	1	2	50	2.3352
**Q14624|ITIH4_HUMAN**	**2**	**3**	**66.667**	**2.905**

**Table 3:** Individual peptides corresponding to proteins classified as strong-evidence candidates (bolded in Table 2). The table lists each peptide along with flanking residues and sequence context, highlighting the fragments that contribute to the protein-level signal.

Protein_ID	Peptides Contributing to Protein Signal
P00450|CERU_HUMAN	K.AETGDKVYVHLK.N, K.VNKDDEEFIESNK.M
P01011|AACT_HUMAN	K.EQLSLLDRFTEDAKR.L, K.ITLLSALVETR.T, R.EIGELYLPK.F
P01023|A2MG_HUMAN	R.TEHPFTVEEFVLPK.F, R.IAQWQSFQLEGGLK.Q, K.FEVQVTVPK.I, K.YGAATFTR.T, K.QQNAQGGFSSTQDTVVALHALSK.Y, K.HYDGSYSTFGER.Y
P01024|CO3_HUMAN	K.LSINTHPSQKPLSITVR.T, R.TKKQELSEAEQATR.T, K.KLVLSSEK.T, R.IPIEDGSGEVVLSR.K, R.VPVAVQGEDTVQSLTQGDGVAK.L, K.SDDKVTLEERLDK.A, K.RIPIEDGSGEVVLSR.K, R.HQQTVTIPPK.S, K.LMNIFLK.D
P02774|VTDB_HUMAN	K.HLSLLTTLSNR.V, K.YTFELSR.R
P02790|HEMO_HUMAN	K.GDKVWVYPPEKK.E, K.GGYTLVSGYPK.R
P04114|APOB_HUMAN	R.NLQNNAEWVYQGAIR.Q, R.TSSFALNLPTLPEVK.F, R.EFQVPTFTIPK.L, K.IEGNLIFDPNNYLPK.E
P06727|APOA4_HUMAN	K.SELTQQLNALFQDK.L, K.SLAELGGHLDQQVEEFR.R
P08603|CFAH_HUMAN	K.SPDVINGSPISQK.I, K.SSNLIILEEHLK.N, K.IDVHLVPDR.K
P0C0L4|CO4A_HUMAN	R.GPEVQLVAHSPWLK.D, K.DHAVDLIQK.G, R.GSFEFPVGDAVSK.V, K.ADGSYAAWLSR.D, R.GLQDEDGYR.M, K.YVLPNFEVK.I
P13645|K1C10_HUMAN	K.SKELTTEIDNNIEQISSYK.S, R.SQYEQLAEQNRK.D, R.ALEESNYELEGK.I
P19823|ITIH2_HUMAN	K.RLSNENHGIAQR.I, K.IQPSGGTNINEALLR.A
Q14624|ITIH4_HUMAN	R.GPDVLTATVSGK.L, R.NVHSGSTFFK.Y

## Data Availability

The raw experimental data are subject to confidentiality restrictions and cannot be shared. Processed and analyzed data supporting the findings of this study are available from the corresponding author upon reasonable request.

## References

[1] National Cancer Institute (NCI) (2024). Cancer Stat Facts: Female Breast Cancer.

[2] American Cancer Society (ACS) (2025). Cancer Facts & Figures 2025.

[3] Centers for Disease Control and Prevention (CDC) (2023). Disparities in Breast Cancer Deaths by Race and Ethnicity, United States, 1999–2020.

[4] Reid S., Cadiz S., Pal T. (2020). Disparities in genetic testing and care among black women with hereditary breast cancer. Curr. Breast Cancer Rep..

[5] Tadi Uppala P., Lum S., Garberoglio C., Uppala G., Kirchner D., Katenhusen R., Kolli K., Mural R., Liebman M. (2008). Shotgun LC/MS proteomics of breast cancer sera from African American women. Cancer Res..

[6] Tadi Uppala P., Garberoglio C., Lum S., Davis W., Leung H.-C.E., Liebman M., Oda K., Patel U.P. (2016). Identification and validation of the potential biomarker insulin-like growth factor binding protein acid-labile subunit for breast cancer in African American women. Cancer Res..

[7] Yadav B.S., Sharma S.C., Chanana P., Jhamb S. (2014). Systemic treatment strategies for triple-negative breast cancer. World J. Clin. Oncol..

[8] Park W.K., Chung S.Y., Jung Y.J., Ha C., Kim J.-W., Nam S.J., Kim S.W. (2024). Long-term oncologic outcomes of unselected triple-negative breast cancer patients according to BRCA1/2 mutations. npj Precis. Oncol..

[9] Zhu Y., Zhu X., Tang C., Guan X., Zhang W. (2021). Progress and challenges of immunotherapy in triple-negative breast cancer. Biochim. Biophys. Acta Rev. Cancer.

[10] Daly B., Olopade O.I. (2015). Race, ethnicity, and the diagnosis of breast cancer. JAMA.

[11] Veyssière H., Bidet Y., Penault-Llorca F., Radosevic-Robin N., Durando X. (2022). Circulating proteins as predictive and prognostic biomarkers in breast cancer. Clin. Proteom..

[12] Iqbal J., Ginsburg O., Rochon P.A., Sun P., Narod S.A. (2015). Differences in breast cancer stage at diagnosis and cancer-specific survival by race and ethnicity in the United States. JAMA.

[13] Keenan T., Moy B., Mroz E.A., Ross K., Niemierko A., Rocco J.W., Isakoff S., Ellisen L.W., Bardia A. (2015). Comparison of the genomic landscape between primary breast cancer in African American versus white women and the association of racial differences with tumor recurrence. J. Clin. Oncol..

[14] Opstal-van Winden A.W.J., Krop E.J.M., Kåredal M.H., Gast M.-C.W., Lindh C.H., Jeppsson M.C., Jönsson B.A.G., Grobbee D.E., Peeters P.H.M., Beijnen J.H. (2011). Searching for early breast cancer biomarkers by serum protein profiling of pre-diagnostic serum; a nested case-control study. BMC Cancer.

[15] Ollberding N.J., Kim Y., Shvetsov Y.B., Wilkens L.R., Franke A.A., Cooney R.V., Maskarinec G. (2013). Prediagnostic leptin, adiponectin, C-reactive protein, and the risk of postmenopausal breast cancer. Cancer Prev. Res..

[16] Srivastava M., Eidelman O., Craig J., Starr J., Kvecher L., Liu J., Hueman M., Pollard H.B., Hu H., Shriver C.D. (2019). Serum biomarkers for racial disparities in breast cancer progression. Mil. Med..

[17] Sickles E.A., D’Orsi C.J., Bassett L.W., Appleton C.M., Berg W.A., Burnside E.S., Feig S.A., Gavenonis S.C., Newell M.S., Trinh M.M. (2013). ACR BI-RADS Mammography. ACR BI-RADS Atlas, Breast Imaging Reporting and Data System.

[18] Luo J., Hippe D.S., Rahbar H., Parsian S., Rendi M.H., Partridge S.C. (2019). Diffusion tensor imaging for characterizing tumor microstructure and improving diagnostic performance on breast MRI: A prospective observational study. Breast Cancer Res..

[19] Jin S., Shen J.-N., Guo Q.-C., Zhou J.-G., Wang J., Huang G., Zou C.-Y., Yin J.-Q., Liu S.-J., Liu W. (2007). 2-D DIGE and MALDI-TOF-MS analysis of the serum proteome in human osteosarcoma. Proteom. Clin. Appl..

[20] Ding Z., Wang N., Ji N., Chen Z.-S. (2022). Proteomics technologies for cancer liquid biopsies. Mol. Cancer.

[21] Zakharova N.V., Bugrova A.E., Indeykina M.I., Brzhozovskiy A.G., Nikolaev E.N., Kononikhin A.S. (2024). The Strategy for Peptidomic LC-MS/MS Data Analysis: The Case of Urinary Peptidome Study. Peptidomics: Methods and Strategies.

[22] Zebene E.D., Lombardi R., Pucci B., Medhin H.T., Seife E., Di Gennaro E., Budillon A., Woldemichael G.B. (2024). Proteomic Analysis of Biomarkers Predicting Treatment Response in Patients with Head and Neck Cancers. Int. J. Mol. Sci..

[23] Sun X., Wang S., Wong C.C. (2024). Mass Spectrometry–Based Proteomics Technology in Pancreatic Cancer Research. J. Pancreatol..

[24] Zhou X., Xue F., Li T., Xue J., Yue S., Zhao S., Lu H., He C. (2024). Exploration of Potential Biomarkers for Early Bladder Cancer Based on Urine Proteomics. Front. Oncol..

[25] Roy R., Schunkert E.M., Olivova P., Gilar M., Geromanos S., Li G.-Z., Gebler J., Dagher A., El-Hayek A., Aldakhlallah R. (2025). Identification of Vitronectin as a Potential Non-Invasive Biomarker of Metastatic Breast Cancer Using a Label-Free LC–MS/MS Approach. Breast Cancer Res..

[26] Karihtala P., Leivonen S.K., Puistola U., Urpilainen E., Jääskeläinen A., Leppä S., Jukkola A. (2024). Serum protein profiling reveals an inflammation signature as a predictor of early breast cancer survival. Breast Cancer Res..

[27] Kaur J., Jung S.Y., Austdal M., Arun A.K., Helland T., Mellgren G., Lende T.H., Janssen E.A.M., Søiland H., Aneja R. (2024). Quantitative proteomics reveals serum proteome alterations during metastatic disease progression in breast cancer patients. Clin. Proteom..

[28] Anderson N.L., Anderson N.G. (2002). The human plasma proteome: History, character, and diagnostic prospects. Mol. Cell. Proteom..

[29] Safari F., Kehelpannala C., Safarchi A., Batarseh A.M., Vafaee F. (2023). Biomarker Reproducibility Challenge: A Review of Non-Nucleotide Biomarker Discovery Protocols from Body Fluids in Breast Cancer Diagnosis. Cancers.

[30] Daly B., Olopade O.I. (2015). A perfect storm: How tumor biology, genomics, and health care delivery patterns collide to create a racial survival disparity in breast cancer and proposed interventions for change. CA Cancer J. Clin..

[31] Guo Y., Zhang H., Yuan L., Chen W., Zhao H., Yu Q.Q., Shi W. (2024). Machine Learning and New Insights for Breast Cancer Diagnosis. J. Int. Med. Res..

[32] Wen X., Guo X., Wang S., Lu Z., Zhang Y. (2024). Breast Cancer Diagnosis: A Systematic Review. Biocybern. Biomed. Eng..

[33] Zaylaa A.J., Kourtian S. (2024). Advancing Breast Cancer Diagnosis through Breast Mass Images, Machine Learning, and Regression Models. Sensors.

[34] Rivera E.C., Johnson J.R., Homan J., Wing S. (2022). Clustering Behavior in Solar Flare Dynamics. Astrophys. J. Lett..

[35] Nordling T.E., Padhan N., Nelander S., Claesson-Welsh L. (2015). Identification of Biomarkers and Signatures in Protein Data. Proceedings of the 2015 IEEE 11th International Conference on e-Science.

[36] Webel H., Niu L., Nielsen A.B., Locard-Paulet M., Mann M., Jensen L.J., Rasmussen S. (2024). Imputation of Label-Free Quantitative Mass Spectrometry-Based Proteomics Data Using Self-Supervised Deep Learning. Nat. Commun..

[37] Lazar C., Gatto L., Ferro M., Bruley C., Burger T. (2016). Accounting for the multiple natures of missing values in label-free quantitative proteomics data sets to compare imputation strategies. J. Proteome Res..

[38] Goeminne L.J.E., Sticker A., Martens L., Gevaert K., Clement L. (2020). MSqRob takes the missing hurdle: Uniting intensity- and count-based proteomics. Anal. Chem..

[39] Breiman L. (2001). Random forests. Mach. Learn..

[40] Bairoch A., Apweiler R., Wu C.H., Barker W.C., Boeckmann B., Ferro S., Gasteiger E., Huang H., Lopez R., Magrane M. (2015). The Universal Protein Resource (UniProt). Nucleic Acids Res..

[41] Zhao Z., Ma J., Mao Y., Dong L., Li S., Zhang Y. (2018). Silence of α1-antitrypsin inhibits migration and proliferation of triple negative breast cancer cells. Med. Sci. Monit..

[42] Shu C., Zha H., Long H., Wang X., Yang F., Gao J. (2020). C3a-C3aR signaling promotes breast cancer lung metastasis via modulating carcinoma associated fibroblasts. J. Exp. Clin. Cancer Res..

[43] Sinha I., Fogle R.L., Gulfidan G., Stanley A.E., Walter V., Hollenbeak C.S., Arga K.Y., Sinha R. (2023). Potential early markers for breast cancer: A proteomic approach comparing saliva and serum samples in a pilot study. Int. J. Mol. Sci..

[44] Varela A.S., Saez J.B.L., Senra D.Q. (1997). Serum ceruloplasmin as a diagnostic marker of cancer. Cancer Lett..

[45] Cordeiro Y.G., Mulder L.M., van Zeijl R.J.M., Paskoski L.B., van Veelen P., de Ru A., Strefezzi R.F., Heijs B., Fukumasu H. (2021). Proteomic analysis identifies FNDC1, A1BG, and antigen processing proteins associated with tumor heterogeneity and malignancy in a canine model of breast cancer. Cancers.

[46] Olbromski M., Mrozowska M., Piotrowska A., Kmiecik A., Smolarz B., Romanowicz H., Blasiak P., Maciejczyk A., Wojnar A., Dziegiel P. (2024). Prognostic significance of alpha-2-macroglobulin and low-density lipoprotein receptor-related protein-1 in various cancers. Am. J. Cancer Res..

[47] Kuhajda F.P., Piantadosi S., Pasternack G.R. (1989). Haptoglobin-related protein (Hpr) epitopes in breast cancer as a predictor of recurrence of the disease. N. Engl. J. Med..

[48] Sakwe A.M., Koumangoye R., Goodwin S.J., Ochieng J. (2010). Fetuin-A (α2HS-glycoprotein) is a major serum adhesive protein that mediates growth signaling in breast tumor cells. J. Biol. Chem..

[49] Santana M.F.M., Caldas Sawada M.I.B.A., Souza Junior D.R., Giacaglia M.B., Reis M., Xavier J., Côrrea-Giannella M.L., Soriano F.G., Gebrim L.H., Ronsein G.E. (2024). Proteomic Profiling of HDL in Newly Diagnosed Breast Cancer Based on Tumor Molecular Classification and Clinical Stage of Disease. Cells.

[50] Fiorito V., Tolosano E. (2022). Hemopexin and cancer. Int. J. Mol. Sci..

[51] Harborg S., Ahern T.P., Feldt M., Rosendahl A.H., Cronin-Fenton D., Melander O., Borgquist S. (2022). Circulating lipids and breast cancer prognosis in the Malmö Diet and Cancer Study. Breast Cancer Res. Treat..

[52] Kollerits B., Gruber S., Steinbrenner I., Schwaiger J.P., Weissensteiner H., Schönherr S., Forer L., Kotsis F., Schultheiss U.T., Meiselbach H. (2024). Apolipoprotein A-IV concentrations and cancer in a large cohort of chronic kidney disease patients: Results from the GCKD study. BMC Cancer.

[53] Broek I.V.D., Sparidans R.W., van Winden A.W.J., Gast M.-C.W., van Dulken E.J., Schellens J.H.M., Beijnen J.H. (2010). The absolute quantification of eight inter-α-trypsin inhibitor heavy chain 4 (ITIH4)-derived peptides in serum from breast cancer patients. Proteom. Clin. Appl..

[54] Kim J., Villadsen R. (2020). The expression pattern of epidermal differentiation marker keratin 10 in the normal human breast and breast cancer cells. J. Histochem. Cytochem..

